# Trends in the diagnosis of variant bladder cancer: a national retrospective cohort analysis

**DOI:** 10.3389/fonc.2026.1807817

**Published:** 2026-05-11

**Authors:** Syed N. Rahman, Kandala Keervani, Xiwen Zhao, Curtis J. Perry, Ping Mu, Darryl Martin, Wei Shen Tan, David G. Hesse, Daniel P. Petrylak, Joshua Warrick, Deepika Kumar, Peter A. Humphrey, Fady Ghali

**Affiliations:** 1 Department of Urology, Yale University School of Medicine, New Haven, CT, United States; 2 Division of Oncology, Department of Medicine, Yale School of Medicine, New Haven, CT, United States; 3 Department of Pathology, Yale University School of Medicine, New Haven, CT, United States

**Keywords:** bladder cancer, micropapillary, plasmacytoid, sarcomatoid, variant

## Abstract

**Introduction:**

The diagnosis of variant bladder cancer (VBC) has significant clinical implications, yet remains a challenge for pathologists and clinicians. Our objective was to characterize the changes in VBC diagnosis over time using a national retrospective cohort.

**Methods:**

The National Cancer Database was queried for all cases of pure urothelial and VBC using International Classification of Disease-O-3 morphologic codes from 2004 to 2021. The diagnosis of each type of VBC was trended from 2004 to 2021 to identify significant differences. This was performed in total and across varying facility types (community cancer program, academic program, comprehensive cancer program, and integrated network) with multivariable regression demonstrating significant associations with VBC diagnosis.

**Results:**

Between 2004 and 2021, 753,880 patients with pure urothelial carcinoma (UC) and 30,884 patients with VBC were identified. The diagnosis of VBC across all bladder cancer cases increased from 3.5% of cases in 2004–2009 (first tertile) to 4.3% of cases in 2016–2021 (third tertile) (*p* < 0.001), a relative 22.5% rise. This was concentrated in micropapillary subtype, sarcomatoid subtype, and neuroendocrine carcinoma (*p* < 0.001). Squamous differentiation did not demonstrate a significant change in diagnosis across time (*p* = 0.20). The increase in VBC diagnosis was noted across all facility types, including both community cancer facilities (3.0% of cases in 2004–2009 to 3.7% of cases in 2016–2021) and academic research facilities (4.4% of cases in 2004–2009 to 7.4% of cases in 2016–2021). Multivariable regression demonstrated a significant association between later year of diagnosis, black race, higher clinical stage, and facility type with diagnosis of VBC (*p* < 0.05).

**Conclusion:**

Our findings suggest that the diagnosis of VBC is increasing nationally over time. The increase in diagnosis is among micropapillary, sarcomatoid, and neuroendocrine subtypes, and is occurring across comprehensive cancer and academic centers, reflecting the growing need for a centralized review.

## Introduction

Variant bladder cancer (VBC) is a heterogenous group of bladder cancers that exhibit distinct histological features and are generally considered more aggressive compared with standard urothelial bladder cancer (UBC) (1). Clinical evidence has suggested that VBC differs from UBC with respect to underlying molecular drivers and therapeutic targets (2). Furthermore, VBC tends to present at a higher clinical stage (3) and has a greater propensity for rapid progression (4). These underlying differences extend to clinical response to therapy, with evidence of VBC having different sensitivities to chemotherapy and immunotherapy (5–8), distinctive patterns of dissemination (7–9), and worsened overall survival compared to UBC (10).

Recognition of VBC on initial diagnosis can have significant implications for patients and is thus of key importance (11). Nevertheless, the pathologic diagnosis can be challenging. Observational studies have noted significant rates of inter-reader variability among pathologists diagnosing VBC (12), as well as under-recognition in community practice (13, 14).

We sought to investigate the trends in the incidence of VBC over time as compared to the incidence of UBC. We hypothesize that VBC diagnosis is on the rise over time and that this trend will be most pronounced in academic and cancer centers, and particularly diagnostically challenging subtypes, reflecting improvement in diagnosis.

## Methods

### Data source

The National Cancer Database (NCDB) includes approximately 70% of all new cancer diagnoses annually from over 1,500 programs participating in the American College of Surgeons Commission on Cancer. We queried bladder cancer cases in the NCDB from 2004 to 2021 to identify histologic urinary bladder cancer (International Classification of Diseases for Oncology, 3rd Edition topography codes C67.0-C67.9). We identified patients with VBC including micropapillary, sarcomatoid, squamous carcinoma (pure), adenocarcinoma (pure), neuroendocrine, and plasmacytoid subtypes based on the codes 8131, 8122, 8070/8051, 8140, 8041/8261, and 8082. Pure urothelial histology was based on code 8120. Our final analytic cohort consisted of 753,880 patients with pure urothelial carcinoma (UC) and 30,884 patients with VBC. We recognize that nomenclature has changed over time, with the World Health Organization most recently recognizing a distinction between divergent differentiation, such as squamous differentiation, and histologic variants, such as micropapillary carcinoma. These distinctions have value, but for the sake of simplicity, we chose to combine them under the umbrella of VBC in the present study. Additionally, as our purpose in this manuscript was to investigate the temporal incidence of non-pure urothelial histology as a whole, including both pure variants such as pure squamous and adenocarcinoma, as well as variant urothelial subtypes, collectively we have referred to them in the form of VBC for this manuscript.

The institutional review board exempted the study from review and waived informed consent because only de-identified records were used in the form of a large retrospective dataset.

### Demographic and clinical variables

We extracted clinical and demographic characteristics including age, sex, race, facility type, clinical T/N staging, insurance status, median income quartile, and treating facility volume. Facility types included community cancer programs, comprehensive community cancer programs, academic research programs, and integrated network cancer programs. Time points were tertiles (2004–2009, 2010–2015, and 2016–2021).

### Study outcomes

The primary endpoint was to identify the change in the incidence of VBC over total bladder cancer diagnosis across time. Secondary endpoints were to identify the change in this incidence across subtypes and across treating facility type.

### Statistical analysis

Descriptive statistics were used to summarize the study population. Continuous variables were reported as median values with interquartile ranges (IQRs), while categorical variables were summarized using counts and percentages. The primary outcome of interest was the probability of being diagnosed of VBC. To assess the temporal trend, univariate logistic regression models with year of diagnosis as the independent variable were applied. We then constructed a multivariable logistic regression model to evaluate the association between year and the odds of VBC, adjusting for patient-level characteristics and facility-level characteristics. Statistical significance was assessed at a two-sided alpha level of 0.05. All analyses were performed using R version 4.4.2 (R Foundation for Statistical Computing, Vienna, Austria)^15^.

## Results

Between 2004 and 2021, we identified 753,880 patients with pure UC and 30,884 patients with VBC in our final analytic cohort. Full descriptive statistics regarding both cohorts can be identified in **Table 1**. The median age was 72 years (64,79) in the pure UC cohort and 71 years (62,79) in the VBC cohort, and the majority of patients in the cohort were Caucasian (96% pure UC and 4% VBC, respectively). Additionally, the total cases increased from 215,504 pure UC and 7,847 VBC in 2004–2009 to 282,644 pure UC and 12,735 VBC cases in 2016–2021. The majority of pure UC diagnoses occurred in comprehensive community cancer programs (311,040 cases), whereas the majority of VBC cases occurred in academic programs (11,974 cases).

**Table 1:** Description of total cohort of bladder cancer cases 2004–2021.

		Is variant	
Characteristic	Overall, *N* = 784,764^1^	No, *N* = 753,880^1^	Yes, *N* = 30,884^1^
Age			
Median (IQR)	72 (64, 79)	72 (64, 79)	71 (62, 79)
Race_new			
Asian/Pacific Islander	14,541 (1.9%)	13,900 (1.8%)	641 (2.1%)
Black	44,557 (5.7%)	41,462 (5.5%)	3,095 (10%)
Other/Unknown	12,378 (1.6%)	11,888 (1.6%)	490 (1.6%)
White	713,288 (91%)	686,630 (91%)	26,658 (86%)
CDCC_TOTAL_BEST			
0	544,839 (69%)	524,210 (70%)	20,629 (67%)
1	153,471 (20%)	147,259 (20%)	6,212 (20%)
2	53,420 (6.8%)	50,972 (6.8%)	2,448 (7.9%)
≥3	33,034 (4.2%)	31,439 (4.2%)	1,595 (5.2%)
MED_INC_QUAR_12			
$38,000–$47,999	157,138 (23%)	150,676 (22%)	6,462 (24%)
$48,000–$62,999	193,049 (28%)	185,624 (28%)	7,425 (27%)
<$38,000	98,196 (14%)	93,605 (14%)	4,591 (17%)
≥$63,000	249,351 (36%)	240,637 (36%)	8,714 (32%)
Unknown	87,030	83,338	3,692
YEAR_OF_DIAGNOSIS			
2004	34,473 (4.4%)	33,389 (4.4%)	1,084 (3.5%)
2005	35,249 (4.5%)	34,073 (4.5%)	1,176 (3.8%)
2006	35,460 (4.5%)	34,202 (4.5%)	1,258 (4.1%)
2007	38,266 (4.9%)	36,920 (4.9%)	1,346 (4.4%)
2008	39,374 (5.0%)	37,917 (5.0%)	1,457 (4.7%)
2009	40,529 (5.2%)	39,003 (5.2%)	1,526 (4.9%)
2010	41,263 (5.3%)	39,760 (5.3%)	1,503 (4.9%)
2011	42,481 (5.4%)	40,811 (5.4%)	1,670 (5.4%)
2012	43,740 (5.6%)	42,029 (5.6%)	1,711 (5.5%)
2013	45,007 (5.7%)	43,284 (5.7%)	1,723 (5.6%)
2014	46,140 (5.9%)	44,335 (5.9%)	1,805 (5.8%)
2015	47,403 (6.0%)	45,513 (6.0%)	1,890 (6.1%)
2016	48,666 (6.2%)	46,539 (6.2%)	2,127 (6.9%)
2017	50,037 (6.4%)	47,996 (6.4%)	2,041 (6.6%)
2018	49,839 (6.4%)	47,796 (6.3%)	2,043 (6.6%)
2019	51,309 (6.5%)	49,216 (6.5%)	2,093 (6.8%)
2020	47,667 (6.1%)	45,440 (6.0%)	2,227 (7.2%)
2021	47,861 (6.1%)	45,657 (6.1%)	2,204 (7.1%)
AJCC_clinical_T			
c1	120,436 (15%)	116,807 (15%)	3,629 (12%)
c2	86,019 (11%)	78,070 (10%)	7,949 (26%)
c3	12,690 (1.6%)	10,901 (1.4%)	1,789 (5.8%)
c4	13,952 (1.8%)	11,812 (1.6%)	2,140 (6.9%)
pa	230,941 (29%)	230,720 (31%)	221 (0.7%)
pi	24,237 (3.1%)	24,019 (3.2%)	218 (0.7%)
Unknown	296,489 (38%)	281,551 (37%)	14,938 (48%)
AJCC_clinical_N			
c0	475,569 (61%)	460,837 (61%)	14,732 (48%)
c1	6,366 (0.8%)	5,480 (0.7%)	886 (2.9%)
c2+	9,746 (1.2%)	8,311 (1.1%)	1,435 (4.6%)
cx	72,199 (9.2%)	68,290 (9.1%)	3,909 (13%)
Unknown	220,884 (28%)	210,962 (28%)	9,922 (32%)
Volume_cat			
Q1 volume	50,607 (6.4%)	48,777 (6.5%)	1,830 (5.9%)
Q2 volume	116,200 (15%)	112,073 (15%)	4,127 (13%)
Q3 volume	200,770 (26%)	193,626 (26%)	7,144 (23%)
Q4 volume	417,187 (53%)	399,404 (53%)	17,783 (58%)
FACILITY_TYPE			
Community cancer program	69,746 (8.9%)	67,735 (9.0%)	2,011 (6.5%)
Comprehensive community cancer program	322,009 (41%)	311,040 (41%)	10,969 (36%)
Academic/Research program	229,479 (29%)	217,505 (29%)	11,974 (39%)
Integrated network cancer program	163,530 (21%)	157,600 (21%)	5,930 (19%)

^1^ *n* (%).

### Diagnosis over time stratified by subtype

**Figure 1:**
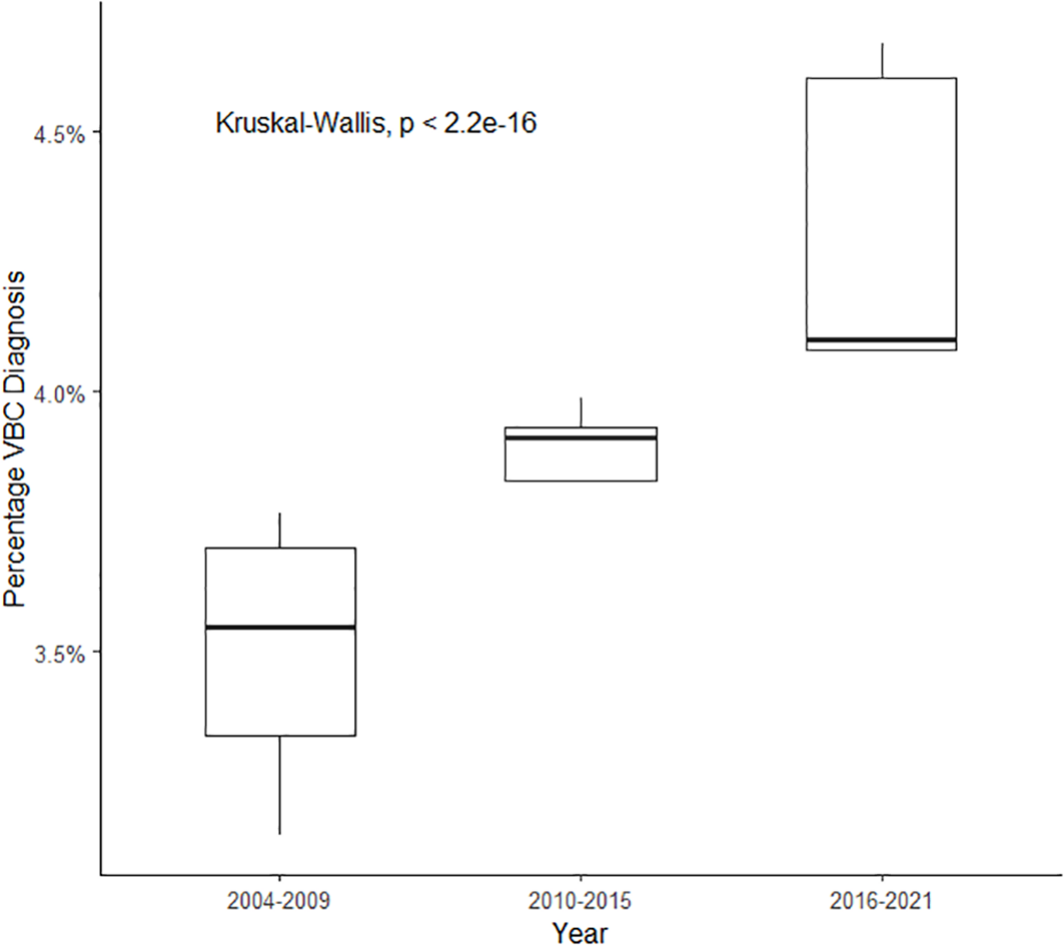
Diagnosis of total VBC across time (percentage of all bladder cancer diagnoses).

**Figure 2:**
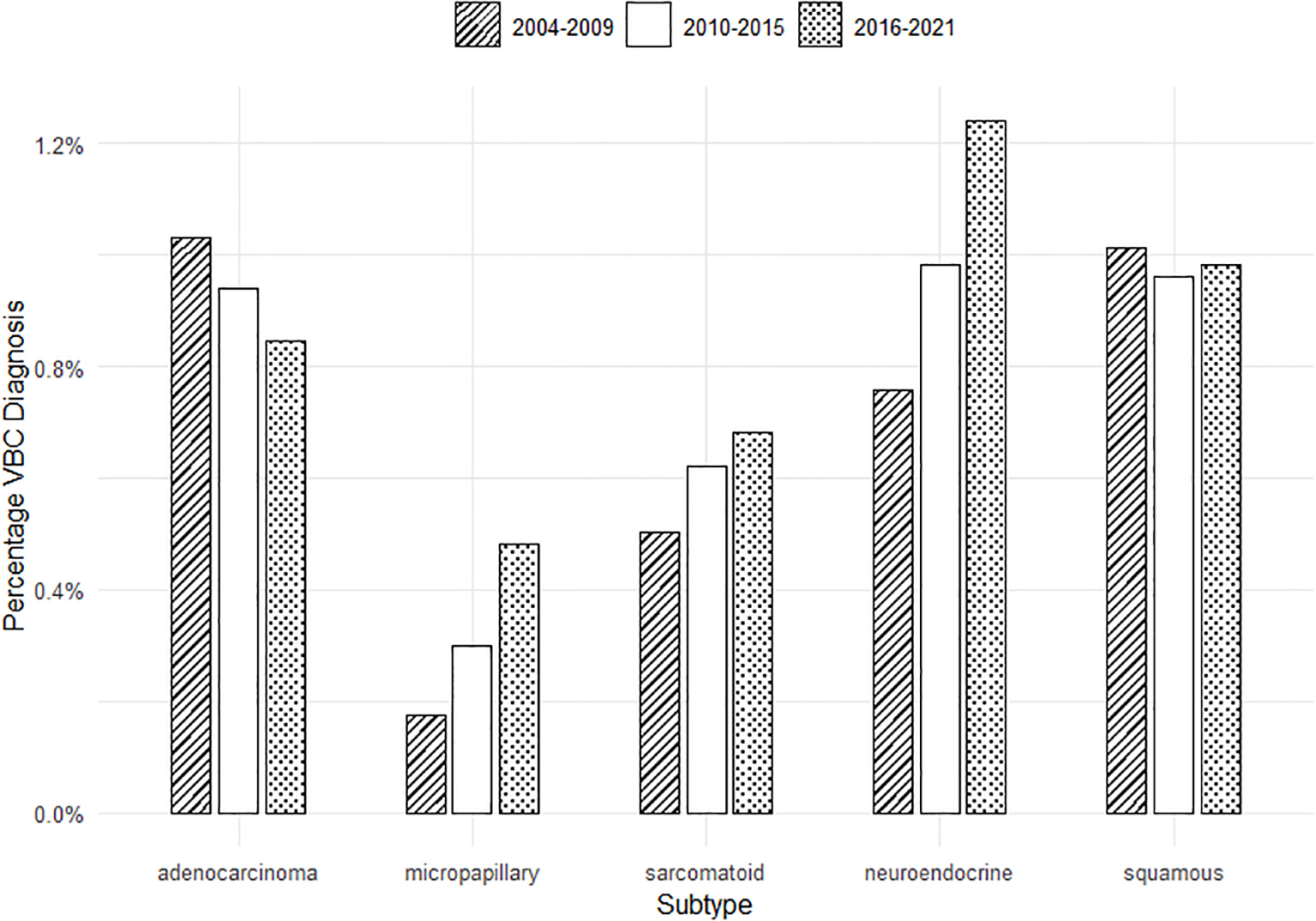
Diagnosis of VBC across time stratified by histologic subtype (percentage of all bladder cancer diagnoses).

In concordance with our hypothesis that the fraction of patients with VBC has increased over time, **Figure 1** demonstrates that the total diagnosis of VBC across all bladder cancer cases increased significantly from 3.5% (SD = 0.21%) of cases in 2004–2009 to 4.3% (SD = 0.25%) of cases in 2016–2021 (*p* < 0.001), a 22.5% relative increase. **Figure 2** reveals the change in diagnosis of VBC over time when stratified by individual subtype/differentiation. Uniquely, adenocarcinoma demonstrates a significant decrease in total diagnosis from 1% of cases in 2004–2009 to 0.8% of cases in 2016–2021 (*p* < 0.001). However, micropapillary, sarcomatoid, and neuroendocrine histologies exhibit a significant increase over this time period. Micropapillary subtype demonstrates a significant increase in cases from 0.2% of cases in 2004–2009 to 0.5% of cases in 2016–2021 (*p* < 0.001), a 150% relative increase. The sarcomatoid subtype shows a significant increase in cases from 0.5% of cases in 2004–2009 to 0.7% of cases in 2016–2021 (*p* < 0.001). The neuroendocrine subtype demonstrates a significant increase in cases from 0.8% of cases in 2004–2009 to 1.2% of cases in 2016–2021 (*p* < 0.001). Squamous differentiation did not significantly change in diagnosis (1% of cases in 2004–2009 to 0.98% of cases in 2016–2021; *p* = 0.14).

### Diagnosis over time stratified by facility type

**Figure 3:**
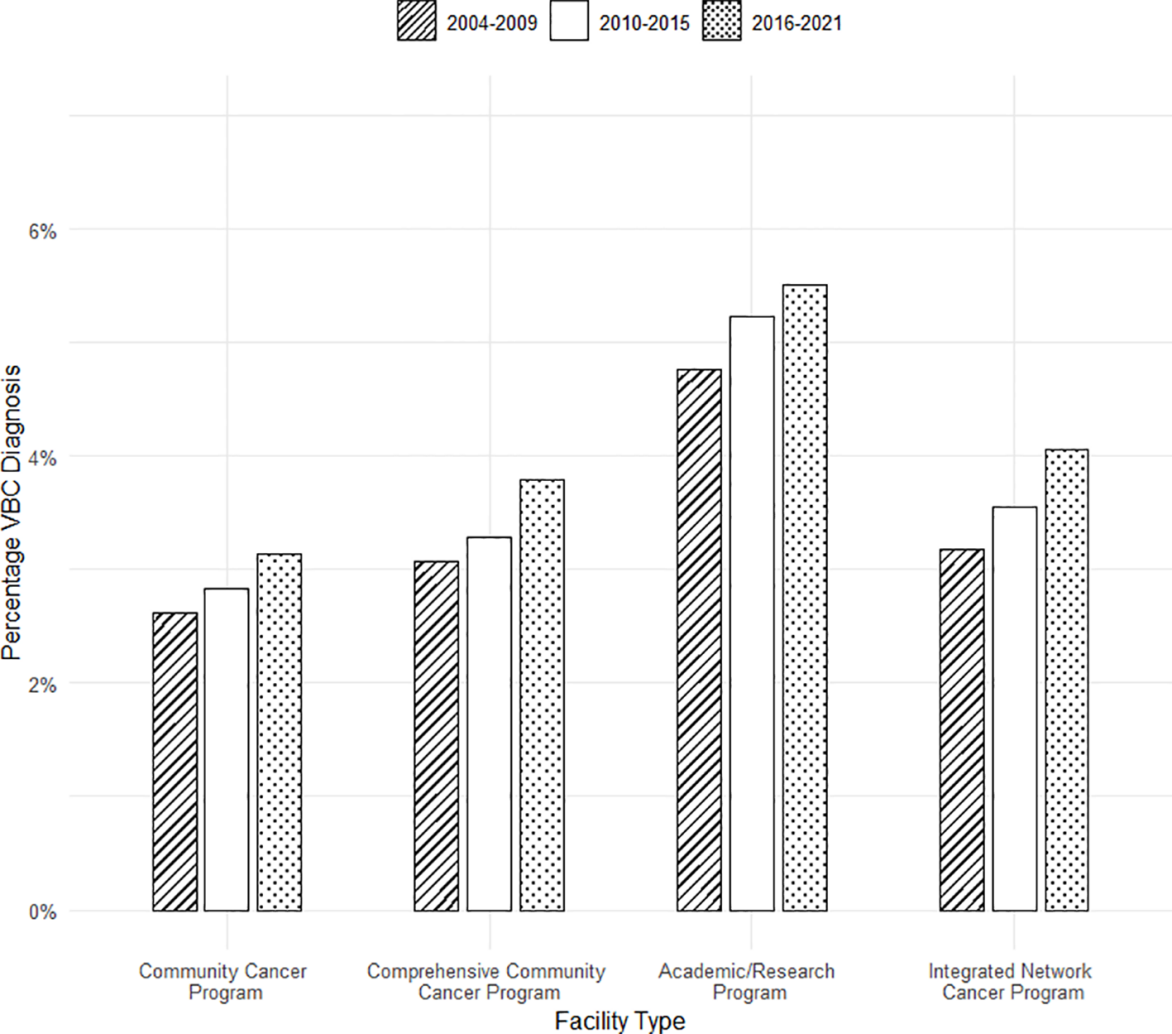
Diagnosis of VBC across time stratified by diagnosing facility type (percentage of all bladder cancer diagnoses).

**Figure 3** establishes the change in diagnosis of VBC over time when stratified by individual diagnosing/treatment facility type. A significant increase in the diagnosis of VBC was noted across all the different facility types over time. In community cancer programs, the diagnosis of VBC increased significantly from 2.6% of cases in 2004–2009 to 3.1% of cases in 2016–2021 (*p* = 0.004). In comprehensive community cancer programs, there was a significant increase in the diagnosis of VBC from 3% of cases between 2004 and 2009 to 3.8% of cases between 2016 and 2021 (*p* < 0.001). In academic/research programs, the diagnosis of VBC increased significantly from 4.8% of cases in 2004–2009 to 5.5% of cases in 2016–2021 (*p* < 0.001). In integrated network cancer programs, the diagnosis of VBC increased significantly as well from 3.2% of cases in 2004–2009 to 4.1% of cases in 2016–2021 (*p* < 0.001).

### Multivariable analysis

**Table 2** exhibits a multivariable regression model that analyzes the correlation between clinical and demographic parameters and the diagnosis of VBC. Increasing age was associated with a lower likelihood of the diagnosis of VBC [OR 0.99 (0.99–0.99) *p* < 0.001]. African American and Asian/Pacific islander races were associated with an increased likelihood of VBC diagnosis [OR 1.51 (1.44–1.58) *p* < 0.001 and 1.12 (1.03–1.22) *p* = 0.009, respectively]. Individuals diagnosed in more recent years had a significantly higher likelihood of diagnosis of VBC [OR 1.02 (1.02–1.02) *p* < 0.001]. Additionally, higher clinical stages had a significantly higher likelihood of VBC diagnosis relative to cT1 (OR 3.15, 4.74, 5.04 *p* < 0.001, cT2–4, respectively). Clinical lymph node positivity also had a significantly higher likelihood of diagnosis of VBC (OR 1.45 and 1.47 *p* < 0.001 for cN1 and c2+, respectively). Facility patient volume was not significantly associated with diagnosis of VBC [OR 1.03 (0.97–1.10) *p* = 0.4 for Q4 volume relative to Q1]. Relative to community cancer programs, comprehensive community cancer programs [OR 1.18 (1.11–1.25) *p* < 0.001], academic research programs [OR 1.46 (1.37–1.55) *p* < 0.001], and integrated network cancer programs [OR 1.24 (1.16–1.31) *p* < 0.001] all had a significantly higher likelihood of VBC diagnosis.

**Table 2:** Multivariate regression model for diagnosis of VBC.

Characteristic	OR*^1^*	95% CI*^1^*	*p*-value
AGE	0.99	0.99, 0.99	<0.001
Race_new			
Asian/Pacific Islander	—	—	
Black	1.34	1.22, 1.48	<0.001
Other/Unknown	0.88	0.77, 1.00	0.047
White	0.89	0.82, 0.97	0.009
MED_INC_QUAR_12			
$38,000–$47,999	—	—	
$48,000–$62,999	0.97	0.94, 1.01	0.11
<$38,000	1.01	0.97, 1.05	0.8
≥$63,000	0.91	0.88, 0.94	<0.001
CDCC_TOTAL_BEST			
0	—	—	
1	1.04	1.01, 1.08	0.010
2	1.12	1.07, 1.17	<0.001
≥3	1.11	1.04, 1.17	<0.001
YEAR_OF_DIAGNOSIS	1.02	1.02, 1.02	<0.001
AJCC_clinical_T			
c1	—	—	
c2	3.15	3.01, 3.28	<0.001
c3	4.74	4.44, 5.06	<0.001
c4	5.04	4.73, 5.36	<0.001
pa	0.03	0.03, 0.04	<0.001
pi	0.30	0.26, 0.34	<0.001
Unknown	2.35	2.23, 2.48	<0.001
AJCC_clinical_N			
c0	—	—	
c1	1.45	1.34, 1.57	<0.001
c2+	1.47	1.38, 1.57	<0.001
cx	0.98	0.94, 1.03	0.5
Unknown	0.57	0.55, 0.61	<0.001
Volume_cat			
Q1 volume	—	—	
Q2 volume	0.98	0.92, 1.05	0.6
Q3 volume	0.97	0.92, 1.04	0.4
Q4 volume	1.04	0.98, 1.10	0.2
FACILITY_TYPE			
Community cancer program	—	—	
Comprehensive community cancer program	1.17	1.10, 1.24	<0.001
Academic/Research program	1.45	1.37, 1.55	<0.001
Integrated network cancer program	1.23	1.16, 1.31	<0.001

^1^ OR, odds ratio, CI, confidence interval.

## Discussion

The diagnosis of VBC carries significant clinical implications for both providers and patients. We present an evaluation of national registry data demonstrating an increasing rate of VBC diagnosis across practice types. We observe that the rise in diagnostic rates is particularly seen in certain individual histologic subtypes (micropapillary, neuroendocrine, and sarcomatoid). While the diagnosis of VBC is highest in academic research programs, a rise in the incidence of VBC diagnosis is seen across all facility types.

We report a 22.5% relative increase in VBC diagnosis over time, from 3.5% to 4.3%. The rising diagnosis rate of VBC is best explained by a growing awareness and recognition of the clinical importance of this distinct group of diseases, rather than a true rise in incidence, though our data here cannot adjudicate this question directly. As the investigation into VBC advances, the understanding of the unique biological behaviors and treatment responses has matured. Heightened awareness and recognition of their clinical implications have made diagnosis more consequential, influencing both treatment decisions and patient outcomes. Future improvements in the diagnosis of VBC are likely to benefit patients by identifying those at particularly high risk and facilitating disease-specific treatment. Artificial intelligence and machine learning, for instance, have begun to play a noticeable role in histopathologic evaluation and may even predict clinical outcomes in UC (15). An additional promising tool is the use of three-dimensional digital scanned pathology, which has been successfully implemented in other malignancies like prostate cancer and has shown promise for improving the ability to quantify adverse pathologic features like Gleason pattern-4 disease or lymphovascular invasion, in a manner that is less compromised by cross-sectioning (16–18).

Accurate pathologic diagnosis of VBC remains a clinical challenge as the diagnosis of histologic subtypes involves a combination of morphologic and immunophenotypic criteria. Identifying micropapillary subtype, for instance, involves recognizing clusters of high-grade carcinoma cells lacking fibrovascular cores and within lacunar spaces, without precise histomorphologic features in invasive cancer, which cannot be confirmed or refuted by immunohistochemistry. In contrast, differentiation to small cell carcinoma is typically confirmed by expression of neuroendocrine markers, such as synaptophysin and chromogranin, and sarcomatoid carcinoma is often supported by expression of cytokeratins and p63, all by immunohistochemistry (18–20). Retrospective analyses have demonstrated the importance of central pathology review at high-volume academic centers for the diagnosis of VBC. In a retrospective analysis of 589 transurethral resection samples referred for academic central review, Shah et al. found that 44% of samples with variant subtypes were not reported by the referring institution (13). Another retrospective analysis by Luchey et al. of 1,191 cases sent from community practices to a comprehensive cancer center found a 27.4% rate of pathologic change on second opinion that ultimately correlated with treatment changes in 15% of cases (14). On multivariable analysis, we find that community cancer programs were less likely to recognize and diagnose variant bladder types compared with comprehensive community cancer programs [OR 1.17 (1.10–1.24), *p* < 0.001], integrated network cancer programs [OR 1.23 (1.16–1.31), *p* < 0.001], and academic/research programs [OR 1.45 (1.37–1.55), *p* < 0.001]. This may reflect referral patterns, yet it aligns with prior reports and underscores the importance of a centralized pathologic review and referral to higher-volume academic centers when VBC is suspected.

The change in VBC diagnosis over time is not uniform across the subtypes, likely reflecting the distinct challenges each poses. Prior studies have reported that while squamous differentiation was the most commonly diagnosed variant subtype, the micropapillary subtype and neuroendocrine type were the most underrecognized by referring institutions (13). Our study similarly finds clear differences in the increasing trends by subtype, with micropapillary, sarcomatoid, and neuroendocrine subtypes experiencing significant increases over time, while adenocarcinoma and squamous subtypes did not. Micropapillary, for instance, was reported at 0.2% in the period of 2004–2009, but increased to 0.5% in 2016–2021, a 150% relative increase, though we note the very small incidence. This observation can further focus future efforts to optimize the diagnostic sensitivity of a VBC pathology review. Furthermore, our results are similar to that of a recent retrospective analysis by Wallace et al. demonstrating overall increases in the proportion of VBC diagnoses but decreases in the diagnosis of the squamous subtype, corroborating the growing awareness in VBC and the need for a centralized review (21).

There are important clinical implications for patients with VBC. This group of diseases is generally associated with poor outcomes compared with urothelial BC and suffers from chemoresistance, higher burden of disease at presentation, and frequent upstaging at the time of radical cystectomy (18–20). Patients with VBC experience varying benefits from standard interventions like neoadjuvant chemotherapy and lymph node dissection at the time of radical cystectomy (16, 18, 22). Our results demonstrating a significant increase in the diagnosis of neuroendocrine, micropapillary, and sarcomatoid histologies represent an increase in the proportion of patients likely to receive subtype-informed clinical care and may lead to more appropriate therapy.

Additionally, there are specific novel findings in our analysis worthy of further elaboration. For instance, there are apparent variants in the diagnosis of certain variants such as the decrease in adenocarcinoma over time. It is important to understand that instead of directly reflexing underlying epidemiologic shifts, this may instead reflect an underlying shift in coding patterns. Moreover, we identified in multivariable analysis an association between African American race and an increase in VBC diagnosis that may reflect underlying differences in access to care and initial age of presentation. Finally, it is important to underscore that ultimately our identified relative increase in the diagnosis of VBC represents a small overall increase in absolute terms. This may partially be due to the prevalence in the NCDB being lower than other institutional series. However, it is important to note that this modest relative increase could intrinsically reflect improved pathologic recognition of variant histologies throughout time as opposed to an inherently increased disease incidence.

An increase in recognition of VBC may reflect practice pattern changes in the diagnosis of bladder cancer. Recent multi-institutional studies demonstrate that the integration of urine-based molecular assays with cytology can improve diagnostic performance and may influence reporting patterns over time (23, 24). Specifically, emerging transcriptomics-based profiling may enhance tumor characterization and directly influence the recognition of VBC as it is already proving beneficial in the detection of muscle invasive bladder cancer as a whole (25).

This study has several important limitations. This is a retrospective analysis of a national database that is prone to errors of data reporting and capture, variations in coding, and limitations of granularity of clinical information. Our study is observational in nature and cannot give causation or explanatory inference. Additionally, we are unable to determine the percentage of subtype on diagnosis and could not account for combinations of histologic subtypes. The source of our data also excluded invasive UC with squamous differentiation. This finding is common in bladder cancer, seen in approximately 20% of invasive tumors^21^. This contrasts with pure squamous cell carcinoma, such as that collected in the present data source, which is substantially less common. Similar studies in datasets with more granular pathologic delineation as well as clinical outcomes will be informative in collaboration with genitourinary pathologists. Additionally, facility-type comparisons in our analysis should be interpreted with caution as the coding in the NCDB may reflect underlying referral patterns and reporting patterns rather than initial diagnostic workup.

## Conclusions

Our findings suggest that the diagnosis of VBC is steadily increasing nationwide over time, largely among micropapillary, sarcomatoid, and neuroendocrine histologies. This increase is occurring across community and academic centers, prompting the growing need for a subspecialized central review by genitourinary pathologists and the recognition of both diagnosis and management of subtypes by pathologists and urologists.

## Data Availability

The raw data supporting the conclusions of this article will be made available by the authors, without undue reservation.
